# Very long‐term survivors among patients with metastatic soft tissue sarcoma

**DOI:** 10.1002/cam4.1931

**Published:** 2019-03-27

**Authors:** Mélodie Carbonnaux, Mehdi Brahmi, Camille Schiffler, Pierre Meeus, Marie‐Pierre Sunyach, Amine Bouhamama, Marie Karanian, Franck Tirode, Daniel Pissaloux, Gualter Vaz, Isabelle Ray‐Coquard, Jean‐Yves Blay, Armelle Dufresne

**Affiliations:** ^1^ Department of Medical Oncology Centre Léon Bérard Lyon France; ^2^ Université Claude Bernard Lyon France; ^3^ Department of Clinical Research Centre Léon Bérard Lyon France; ^4^ Department of Surgery Centre Léon Bérard Lyon France; ^5^ Department of Radiology Centre Léon Bérard Lyon France; ^6^ Department of Biopathology Centre Léon Bérard Lyon France; ^7^ Department of Translational Research and Innovation Centre Léon Bérard Lyon France

**Keywords:** clinico-pathological characteristics, long‐term survivors, metastatic sarcomas, prognostic factors, soft tissue sarcomas

## Abstract

**Background:**

Metastatic soft tissue sarcomas (STS) are a group of rare and heterogeneous mesenchymal tumors with a poor prognosis. The aim of this study was to evaluate the incidence of long‐term survivors and describe their presentation and management in a large cohort of patients with metastatic STS.

**Methods:**

We collected information of patients with metastatic STS managed in Centre Leon Berard between 1985 and 2015 aiming to compare the group of patients alive 5 years after the diagnosis of metastases vs the others. Prognostic factors of patients and tumors characteristics were investigated by logistic regression analysis. For “long‐term survivors,” we explored therapeutic strategies at metastatic stage.

**Results:**

Out of 436 patients enrolled, 39 (9%) were still alive 5 years after diagnostic of metastases with a median survival of 146 months (12 years). This “long‐term survivors” group included more female and younger patients, with better performance status, more synovial sarcoma or endometrial stromal sarcoma, more patients with simple genomic sarcomas, lower tumor grade, smaller tumor, and longer disease‐free interval. In multivariate analysis, age below 55 at metastatic stage (*P* = 0.0002) and grade 1 tumor (*P* < 0.0001) were significantly associated with the “long‐term survivors.” Their therapeutic management was usually aggressive (intensified or polychemotherapy, repeated local treatment of metastases), leading to 62% of complete response in first‐line setting.

**Conclusions:**

Very long‐term survivors are observed in metastatic STS. Selection of patients in good condition with less aggressive tumor and administration of intensive treatment may lead to obtain these motivating results in a poor prognosis disease.

## 1 INTRODUCTION

Soft tissue sarcomas (STS) are a group of rare and heterogeneous tumors with more than 70 different histological subtypes identified, usually classified between simple and complex genomic sarcomas. The overall annual incidence is estimated to 5.6‐5.9 per 100 000 adults in Europe. The prognosis of this disease is in favor of localized disease assuming reliable diagnostic procedures and a careful initial surgery according to clinical practice guidelines. Unfortunately, nearly half of patients with STS develop distant metastases. The median range of overall survival (OS) for metastatic STS patients is between 12 and 18 months, with less than 20% of patients still alive at 2 years.^1^

Many prognostic factors have demonstrated a significant impact on survival in patients with metastatic STS, such as age at diagnostic, gender, performance status (PS), histological subtype, grade, localization and size of tumor, site and number of metastasis, lymphopenia and others inflammatory biomarkers.^2^, ^3^, ^4^, ^5^, ^6^ Therapeutic management, compliance to clinical practice guidelines, and sensitivity to treatment are also prognostic indicators of high value.

The goal of treatment in metastatic STS setting is considered as palliative and chemotherapy is usually administrated to control tumor growth despite a poor improvement in survival. Doxorubicin is the most efficient drug with a 10%‐14% response rate in first‐line setting and a median progression‐free survival (PFS) of 4.5 months.^7^ Doxorubicin‐based combination improves response rate and extends progression‐free survival (PFS) but fails to prolong OS.^1^ Second‐line treatment and beyond are based on histology‐driven choice.

Surgical resection of lung metastases, if complete excision of all lesions is feasible and in lack of extrapulmonary disease is considered a standard treatment in the last ESMO‐EURACAN recommendations.^8^ Indeed, surgical strategy has been associated with long‐term survival in retrospective case‐series, with 5‐year survival rates ranging from 15% to 50.9%.^9^

The objectives of this study were to evaluate the frequency of long‐term survivors in patients with metastatic STS, to describe their clinico‐pathological characteristics and modalities of their therapeutic management in order to identify prognostic factors for OS.

## 2 METHODS

### 2.1 Study design and population

MetaSarc database prospectively included patients with metastatic STS managed in one of the three national reference centers designated by the French National Center Institute (Centre Léon Bérard, Lyon; Institut Bergonié, Bordeaux; and Institut Gustave Roussy, Villejuif). From this national database, this study included patients above 18 years old with metastatic STS fully managed at the Centre Leon Berard between 1985 and 2015. Patients with gastrointestinal stromal tumors were excluded. “Long‐term survivors” were defined as patients alive 5 years after the diagnosis of a metastatic disease. Patients with no follow‐up available at 5 years after inclusion were not considered in this analysis.

Patient information and tumor characteristics were gathered from the MetaSarc database and completed with data of electronic medical records. The distribution of patients’ characteristics was compared between the long‐term survivor group and the group of patients who died within 5 years after metastatic diagnosis, defined as control group. For long‐term survivors only, treatment administered at metastasis stage was described.

Overall survival, PFS, disease‐free interval (DFI), and response to treatment were investigated. OS was defined as the time between metastatic diagnosis and date of death or censored to date of last contact. PFS was defined as the time from treatment initiation to the date of event defined as the first progression or death due to any cause or censored to date of last contact. The DFI was defined as the time interval between resection of primary tumor and diagnosis of metastases. Responses were defined according to the RECIST v1.1 criteria.^10^

### 2.2 Statistical analysis

Categorical data were summarized by frequencies and percentages, the continuous covariates by median with range. The clinico‐pathological characteristics of the patients were compared between the long‐term survivor and control groups using the chi‐square test or Fisher exact for categorical data and Wilcoxon test for continuous variables. Survival curves were estimated using the Kaplan‐Meier technique. Median follow‐up was calculated using reverse Kaplan‐Meier estimation. We performed a logistic regression analysis to evaluate parameters correlated with 5‐years survival. Variables sufficiently informed (less than 15% missing value) and significant at a 5% level in univariate model were included in a backward selection procedure to keep factors significant at a 5% level in the final. Odds Ratio (OR) is presented with 95% confidence interval (CI). A *P*‐value <0.05 was considered statistically significant. Statistical Analysis Software version 9.4 (SAS Institute, Cary, NC) was used for statistical analysis.

## 3 RESULTS

Two thousand one hundred and sixty‐five patients were referenced in MetaSarc database.^11^ In Centre Leon Berard center, 630 patients with metastatic STS were eligible, and 194 patients with a follow‐up shorter than 5 years were excluded from the analysis. Consequently, 436 patients referred to the Centre Leon Berard between 1985 and 2015 were analyzed and defined as “the whole cohort.” Thirty‐nine patients (9%) were alive 5 years after metastatic diagnostic and defined as “long‐term survivors.” The other ones, dead within 5 years after the diagnosis of metastases were defined as the “control group.”

**Figure 1:**
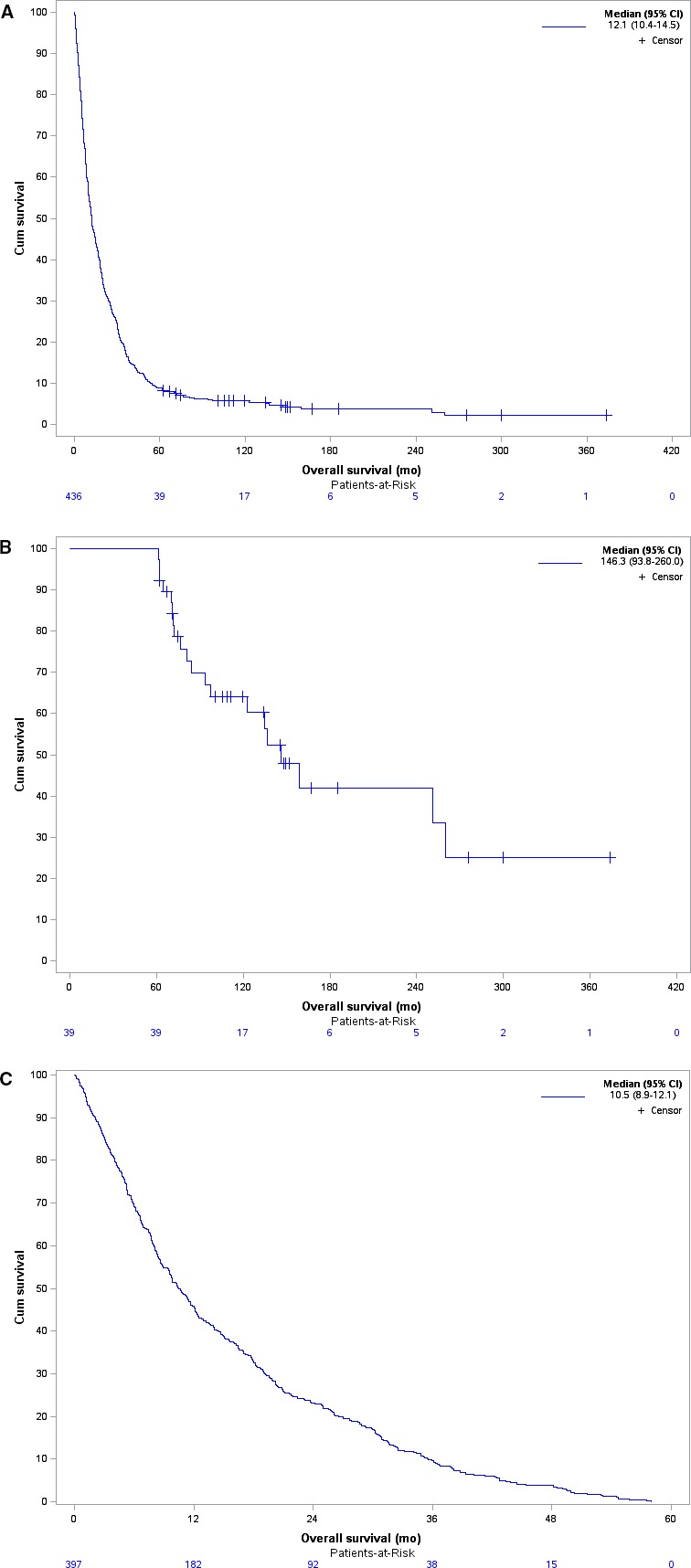
Kaplan‐Meier survival curve illustrating the median overall survival (OS) for (A) the whole cohort of included patients (N = 436 patients), (B) long‐term survivors metastatic STS patients (N = 39 patients) and (C) short‐term survivors (N = 397 patients)

In the whole cohort, the median follow‐up was 149.6 months [95% CI: 119.3‐275.8], the median OS was 12.1 months [95% CI: 10.4‐14.5]. The median OS was 146 months (ie, 12 years) [95% CI: 93.8‐260] for long‐term survivors and 10.5 months (95% CI: 8.9‐12.1] for the control group (Figure 1).

### 3.1 Characteristics of patients and tumors

Patients’ and tumors’ characteristics at baseline are reported in Table 1. Gender was significantly different between groups (*P* = 0.020). Two‐thirds of long‐term survivors were females whereas a predominance of male was observed in the control group. The median age at metastatic stage was significantly lower in long‐term survivors: 46 years [21‐77] vs 59 years [18‐92] (*P* < 0.001). PS at metastatic stage was significantly better in long‐term survivor group (*P* = 0.008), with a PS 0‐1 in 98% of cases.

**Table 1:** Patients’ and tumors’ characteristics

Characteristics	Long‐term survivors (n = 39) N (%)	Control group (n = 397) N (%)	*P* value
Patients’ characteristics			
Gender			
Male	13 (33.3)	210 (52.9)	
Female	26 (66.7)	187 (47.1)	0.020
Age at metastasis stage			
Median in years [range]	46 [21‐77]	59 [18‐92]	<0.001
PS at metastasis stage			
0	26 (66.7)	86 (43)	
1	12 (30.8)	81 (40.5)	
≥2	1 (2.6)	33 (16.5)	
Missing	0	197	0.008
Tumors’ characteristics			
Tumor localization			
Extremity	16 (41)	144 (36.3)	
Viscera/Retroperitoneum	18 (46.2)	153 (38.5)	
Trunk	5 (12.8)	46 (11.6)	
Other	0 (0)	54(13.6)	0.045
Histology			
Leiomyosarcoma	8 (20.5)	21.4)	
Synovial sarcoma	6 (15.4)	32 (8.1)	
UPS	5 (12.8)	100 (25.2)	
Endometrial stromal sarcoma	5 (12.8)	1 (0.3)	
Liposarcoma	3 (7.7)	51 (12.8)	
Others^a^	12 (30.8)	128 (32.2)	<0.001
Size of tumor			
≤5 cm	13 (36.1)	68 (19.3)	
>5 cm	23 (63.9)	284 (80.7)	
Missing	3	45	0.018
Grade of tumor			
G1	13 (33.3)	18 (4.9)	
G2	13 (33.3)	130 (35.1)	
G3	13 (33.3)	222 (60)	
Missing	0	27	<0.001
Tumor depth^b^			
Deep	36 (92)	345 (86.9)	
Superficial	3 (8)	32 (8.1)	
Superficial and deep	0 (0)	20 (5)	0.463
Multifocal tumor			
Yes	6 (15)	23 (22.5)	
No	33(85)	79 (77.5)	
Missing	0	295	0.346
Sarcoma genomics			
Simple genomic sarcoma	21 (53.8)	134 (33.8)	
Complex genomic sarcoma	18 (46.2)	263 (66.2)	0.012
Simple genetic alteration			
Translocation	19 (90.5)	88 (65.7)	
Amplification	1 (4.8)	42 (31.3)	
Activating mutation	1 (4.8)	1 (0.7)	
Inactivating mutation	0 (0)	3 (2.2)	0.017
Stage of disease			
Metastatic disease at diagnosis	14 (35.9)	112 (8.2)	
Metastatic relapse	25 (64.1)	285 (71.8)	0.312
DFI (mo)			
≤24	25 (64)	325 (82)	
>24	14 (36)	72 (18)	0.008
Number of metastatic sites			
1	31 (79.5)	257 (76.5)	
≥2	8 (20.5)	79 (23.5)	
Missing	0	61	0.674
Liver metastasis	3 (7.7)	88 (22.2)	
No liver metastasis	3 (92.3)	309 (77.8)	0.034

DFI, disease‐free interval; PS, Performance status; UPS, Undifferentiated pleomorphic sarcoma.

a Others: Angiosarcoma, Alveolar soft part sarcoma, Atypical lipomatous tumor, Clear cell sarcoma, Chondrosarcoma, Dermatofibrosarcoma protuberans, Desmoplastic round cell tumor, Ewing sarcoma, Epithelioid haemangioendothelioma, Epithelioid sarcoma, Fibromyxoid sarcoma, Inflammatory myofibroblastic tumor, Intimal sarcoma, Malignant hemangiopericytoma, Malignant solitary fibrous tumor, Malignant Peripheral Nerve Sheath Tumor, Myoepithelioma, Myxofibrosarcoma, Osteosarcoma, Rhabdomyosarcoma, Sclerosing epithelioid fibrosarcoma, PECOMA, Rhabdoid tumor, Rhabdomyosarcoma.

b Superficial tumor is located exclusively above the superficial fascia without invasion of the fascia; deep tumor is located either exclusively beneath the superficial fascia, superficial to the fascia with invasion of the fascia, or both superficial yet beneath the fascia*.*

Primary tumor localization was statistically different between groups (*P* = 0.045) with more frequent localization of extremity, retroperitoneum, or viscera in long‐term survivors. Distribution of histological subtypes was significantly different in the two groups (*P* < 0.001). Low‐grade endometrial stromal sarcoma (13%) was exclusively represented in long‐term survivor group, and synovial sarcoma (15% vs 8%) was frequently represented in long‐term survivor group, in contrast to undifferentiated pleomorphic sarcoma (UPS) (13% vs 25%) and liposarcoma (8% vs 13%) more represented in the control group. Among liposarcoma, there was no histology subtype trend in long‐term survivors, in contrast to control group in which dedifferentiated and pleiomorphic liposarcoma clearly predominated (more than 90%). Leiomyosarcoma was highly represented (about 20%) in the two groups without difference.

Median size of primary tumor differed between groups (*P* = 0.018) with a less frequent proportion of large tumor >5 cm in long‐term survivors (64% vs 81%). Distribution of tumor grade significantly differed between groups (*P* < 0.001). Grade 1, 2, and 3 tumors were equally represented (33%) in long‐term survivors whereas grade 3 tumor widely predominated in the control group (60%).

Biological classification differed between groups (*P* = 0.012), with a majority of simple genomic sarcoma (54%) in long‐term survivors and a majority of complex genomic sarcoma (66%) in the control group. Among patients with a simple genetic alteration, translocation alterations were more frequent in long‐term survivors (90% vs 65%), as well as activating mutation (5% vs 0.7%, respectively); in contrast to amplification (5% vs 31%) and inactivating mutation (0% vs 2%; *P* = 0.017).

The stage of the disease at diagnosis did not differ in the analyzed population with metastatic disease at diagnosis in around 1/3 of patients. Proportion of patients with longer DFI (>24 months) was more frequent in the long‐term survivor group than in the control group (36% vs 18%, respectively, *P* = 0.0096). In the whole cohort, the most frequent localization of first metastases was lung (>50%). No cerebral metastasis was reported. The number and location of metastatic sites did not significantly differ between groups besides liver metastases which are less frequent in long‐term survivors (8% vs 22%, respectively, *P* = 0.034).

### 3.2 Prognostic factor for OS

The univariate analysis (Table 2) identified female gender, age at metastatic stage below 55 years old, good PS, histological diagnosis of ESS, simple genomic profile, tumor size below 5 cm, low grade of tumor, DFI longer than 24 months, and lack of liver metastasis as significant good prognostic factors.

**Table 2:** Univariate and multivariate analyses of prognostic factors for 5‐y survival of 436 patients (409 with complete covariate information)

Characteristics	Univariate analysis		Multivariate analysis	
	Odds Ratio [95% CI]	*P* value	Odds Ratio [95% CI]	*P* value
Gender				
Female	1			
Male	0.445 [0.22‐0.89]	0.0224	‐	‐
Age at metastasis stage (y)				
≤ 55	1			
> 55	0.291 [0.14‐0.6]	0.0009	0.229 [0.1‐0.5]	0.0002
PS at metastasis stage				
0	1			
1	0.490 [0.23‐1.04]			
≥2	0.100 [0.01‐0.77]	0.0240	‐	‐
Histology				
Synovial sarcoma	1			
Endometrial stromal sarcoma	26.656 [2.63‐270.5]			
Leiomyosarcoma	0.502 [0.16‐1.56]			
Liposarcoma	0.314 [0.07‐1.34]			
Others	0.500 [0.17‐1.43]			
UPS	0.267 [0.08‐0.93]	0.0031	‐	‐
Sarcoma genomics				
Simple genomic sarcoma	1			
Complex genomic sarcoma	0.437 [0.22‐0.85]	0.0143	‐	‐
Size of tumor				
≤5 cm	1			
>5 cm	0.424 [0.2‐0.88]	0.0211	‐	‐
Grade of tumor				
1	1		1	
2	0.138 [0.06‐0.35]		0.125 [0.05‐0.33]	
3	0.081 [0.03‐0.2]	<0.0001	0.065 [0.02‐0.17]	<0.0001
Liver metastasis	1			
No liver metastasis	3.417 [1.03‐11.36]	0.0450	‐	‐
DFI (mo)				
≤24	1			
>24	2.528 [1.25‐5.10]	0.0096	‐	‐

DFI, disease‐free interval; PS, Performance status; UPS, Undifferentiated pleomorphic sarcoma.

The multivariate analysis (Table 2) performed on a subset of 409 patients with all necessary information available identified grade 1 tumor (*P* < 0.001) and young age at metastasis stage (*P* = 0.0002) as independent prognostic factors for OS.

### 3.3 Therapeutic management of 39 long‐term survivors

We analyzed in details therapeutic management of the long‐term survivors. Regarding systemic treatment, 28 patients (74%) have been treated by chemotherapy in first‐line metastatic approach, using more frequently combined regimens (77%) compared to monotherapy (20%) or hormonal therapy (3%). Doxorubicin‐containing regimens were administrated to 26 patients (90%). Intensification strategy using high‐dose (HD) chemotherapy was performed in five patients.

Eighteen patients (46%) received 5 or more lines of treatment and 8 of them (20%) received 8 or more lines of treatment, usually including enrollment in clinical trials. We observed that 23 patients alive at 5 years (59%) were enrolled in a clinical trial in metastatic setting (NCT01771458, NCT01494688, NCT00796120, NCT00410462, ET‐743, EORTC 62043, PALETTE ‐ VEG110727, PALSAR, EORTC 62043, BP29428, EORTC‐62012, AP23573 ‐ ARIAD, SUCCEED, REGO‐SARC1214, IMCLONE CP13‐0707, NP27872, EFC 10145).

The use of locoregional treatment for primary tumor and for metastatic sites was analyzed. Fourteen patients among long‐term survivors had a metastatic disease at diagnosis. Seventy‐one percent of them had their primary tumor resected despite synchronous metastases. Finally, 100% of primary tumor management resulted in locoregional control.

**Figure 2:**
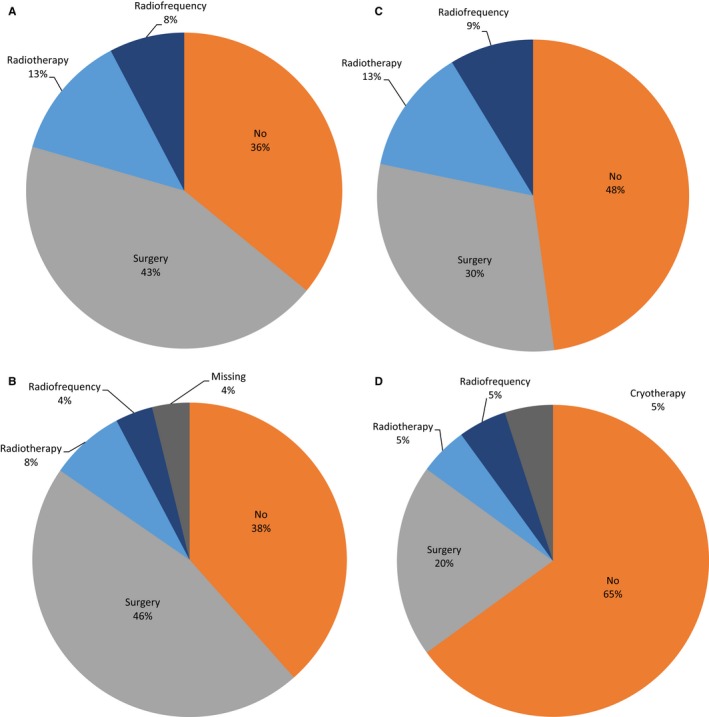
Locoregional modalities of metastatic site in first four lines of treatment for long‐term survivors The use of locoregional treatment (surgery, radiotherapy, radiofrequency, or cryotherapy) in successive metastatic lines: (A) First, (B) Second, (C) Third, and (D) Fourth metastatic line

Sixty‐four percent of long‐term survivors benefited from a locoregional treatment (surgery, radiotherapy, radiofrequency or cryotherapy) of metastatic sites in first‐line setting. Moreover, the use of locoregional treatment was observed in 48%, 52%, and 35% of patients in second, third, and fourth line of treatment, respectively, (Figure 2) leading to a complete response (CR) rate of 52%, 35% and 20% in 2nd, 3rd, and 4th lines, respectively.

**Figure 3:**
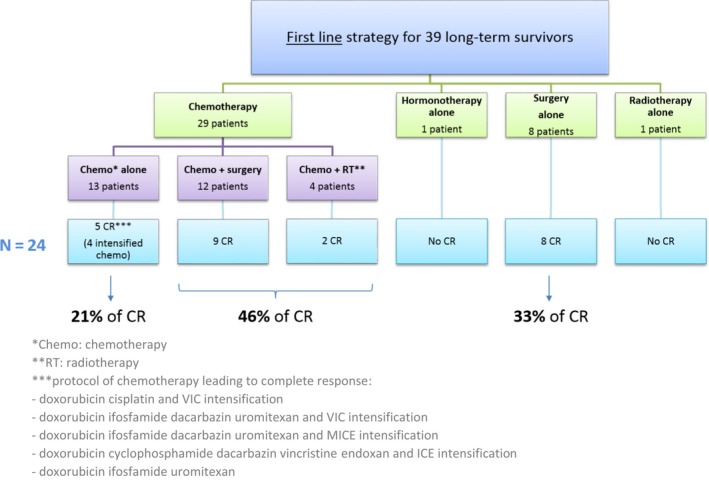
Therapeutic strategy in first line for long‐term survivors (N = 39) and modalities leading to complete response (CR) (N = 24)

Best response to first‐line therapy included 24 (62%) CR, 3 (8%) partial response, and 8 (20%) stable disease. Only four patients (10%) progressed during the first therapeutic line. Response to first‐line and therapeutic strategy leading to CR were explored (Figure 3). Out of 24 patients with CR, five (21%) were obtained with chemotherapy alone, 11 (46%) with combined treatment (chemotherapy and surgery or radiotherapy), and 8 (33%) with surgery alone. The treatment based on chemotherapy alone leading to CR used mainly exclusively intensification protocols.

The median PFS was 30.5 months [17.4‐56.9 months], 19.6 months [11.3‐36.0 months], 9.7 months [4.3‐15.0 months], and 8.1 months [5.8‐11.1 months] in patients treated in first, second, third, and fourth lines metastatic disease, respectively.

## 4 DISCUSSION

Patients with metastatic STS are rare and present poor prognostic. However, this series shows that 9% of them are still alive 5 years after initial metastatic diagnosis and are even very long‐term survivors, with an unexpected prolonged median OS of 12 years. Therefore, analysis of prognostic factors and predictive factor of response to treatment must be explored to adjust the therapeutic strategy according to patient's characteristics.

Regarding clinical characteristics in our series, we observed more female in the “long‐term survivors” group (female to male ratio of 2/1) than in the control group which may be partly explained by a higher frequency of low‐grade endometrial stromal sarcoma (13%) known to be a histological characteristic of less aggressive tumors. Long‐term survivors were also younger (median age: 43 years) and had a good PS (PS 0‐1 for 98%). Besides being recognized as independent parameters correlated to prolong survival,^12^ age and PS are clinical characteristics allowing more aggressive therapeutics (polychemotherapy, HD chemotherapy, successive surgical resections, and innovative therapies through clinical trial inclusions).

Histological tumor subtypes were differently distributed between the two groups. Low‐grade endometrial stromal sarcoma was more frequent in long‐term survivors, consistently with the predominance of female gender. Synovial sarcoma was also more represented in long‐term survivors and are known to be of better prognostic, with higher sensitivity to chemotherapy.^4^ Dedifferentiated and pleomorphic liposarcoma subtypes clearly predominated in control group, as well as undifferentiated pleomorphic sarcomas known to be poor prognosis disease. Blay et al^12^ demonstrated that histological analysis represented an important prognostic factor for OS during the first 5 years and could lose its prognostic value afterward. In our analysis, histological subtype was not correlated with OS in multivariate analysis.

Tumor grade is a factor highly correlated with prolonged survival in literature and was the most significant prognostic factor in the multivariate analysis of our study. Usually, grade 3 tumor prevails in the distribution of metastatic STS, between 27% and 58%^2^, ^4^, ^5^, ^13^, ^14^ contrary to grade 1 tumor found in 8%‐22% of patients. In our series, tumor grade 1 was much more frequent in long‐term survivors (33% vs 5%, *P* < 0.001).

Also, we noted a significant predominance of simple genomic sarcoma in long‐term survivor (54%) and, more specifically, of translocation‐related sarcoma (90%) especially SSX‐SS18 or JAZF1 fusion transcript, consistently with frequent synovial sarcomas and endometrial stromal sarcomas.

Patients with a long‐time lapse between management of the primary tumor and occurrence of metastasis have a longer OS.^14^ In our analysis, more long‐term survivors had a long DFI (>2 years) than control group (36% vs 18%, *P* = 0.008) and DFI was correlated to OS in univariate analysis. Liver metastasis was significantly less represented in long‐term survivor group, consistently with literature.^12^

“Aggressiveness” of therapeutic strategy at metastatic stage aimed at leading to a complete response in first‐line treatment. In literature, this percentage ranged between 20%‐30% in series involving surgery and/or chemotherapy.^12^ Blay et al reported the CR after first‐line treatment being an essential prognostic factor for survival. In our series, one can note a high frequency of CR after first‐line treatment (62%) combining several strategies in our long‐term survivor population: successive ablative treatment, polychemotherapy or HD chemotherapy, and access to innovative treatment through clinical trial.

Surgical resection of primary tumor and metastatic sites was commonly performed in our group of long‐term survivors. Although therapeutic modalities of primary tumor are missing for majority of patients in the control group, one can highlight a very important proportion of patients benefiting from surgical removal of the primary tumor even at metastatic stage. In long‐term survivor group, more than 70% of CR was obtained in first line thanks to ablative treatment (surgery or radiotherapy), alone (33% of CR) or combined to chemotherapy (46% of CR). Ablative techniques were very frequently performed, even in second (62%), third (52%), and fourth (35%) metastatic lines. Despite the lack of randomized trials, recent surgical retrospective series indicates that pulmonary metastasectomy or other ablative treatment would be a valid treatment option to improve the 5‐year survival rates from 15% to 50.9%, mainly in patients with favorable prognostic factors.^10^

Moreover, more than 20% of CR were also obtained after chemotherapy alone in first metastatic line. This chemotherapy was anthracycline‐based in all cases, included in a HD regimen in five patients. However, the routine use of polychemotherapy in metastatic STS is contested. Associations of doxorubicin to various efficient chemotherapy drugs did not result in OS benefit except with olaratumab. However, these combinations improved PFS and tumor response, a clinically important outcome in patient with locally advanced and symptomatic STS inoperable in first intention, but whose tumor could be resectable after sufficient size reduction.^4^, ^15^, ^16^ Histology is also associated with polychemotherapy efficacy. For instance, synovial sarcoma which is highly represented in our series is described like a favorable predictive factor response in case of doxorubicin‐ifosfamide combination regimen.^4^

Five patients benefited fromdose‐intensive chemotherapy with autologous bone marrow transplantation (VIC regimen, ICE regimen, or MICE protocol in PALSAR02 trial) and were in CR Even if prolonged survival has been suggested in phase II trial assessing HD chemotherapy,^17^ these results have never been confirmed in randomized phase III trials comparing HD chemotherapy to conventional dose^15^, ^18^ justifying the interruption of this strategy. However, HD treatment may offer some benefit to a highly selected subgroup of patients.

More than one in two long‐term survivor patients were included in clinical trial, allowing patients to receive new agent before their authorization, such as pazopanib for PALETTE trial. Given the poor prognostic and the rarity of sarcomas, the effort in initiating and running clinical trial is needed to facilitate development and access to new strategy.

Finally, an important finding from this study is that long‐term survivors were observed in all subgroup of patients, even in subgroup with unfavorable clinico‐pathological characteristics such as: older patient (15% with more than 60 years), high‐grade tumors (33% with G3), synchronous metastasis, liver or bone metastasis. Moreover, long‐term survivors were observed even if case of poor response to initial treatment (28% of local recurrence) or to first‐line metastatic treatment (10% of progression disease).

Our series has several limitations. First, data concerning modalities of treatment of the control group are missing, not allowing for comparative analysis. Second, because of the rarity and the aggressiveness of sarcoma, our series included a few numbers of long‐term survivor patients, responsible of low power study. Finally, other classical prognostic factor such as biological parameters (lymphocyte count, neutrophil‐to‐lymphocyte ratio, inflammatory biomarkers, etc), tumors’ characteristics (for instance number of metastasis), and therapeutic management (such as integration of palliative care) were not available for all patients and could not be analyzed.

## 5 CONCLUSION

Metastatic STS remains an incurable disease, but a significant proportion of patients can achieve long‐term survival and even a very long‐term survival with a 12 years median overall survival. Administration of intensive treatment combining systemic therapy and locoregional destruction of metastases in selected young patients with good performance status and slowly progressive disease may lead to obtain these motivating results in a poor prognosis disease. Identification of such factors is essential for patient management and may contribute further to a more individualized strategy of metastatic STS patients.

## CONFLICT OF INTEREST

The authors made no disclosures.

## References

[1] ESMO/European Sarcoma Network Working Group . Soft tissue and visceral sarcomas: ESMO Clinical Practice Guidelines for diagnosis, treatment and follow‐up. Ann Oncol. 2014;25(Suppl 3):iii102‐112. 25210080 10.1093/annonc/mdu254

[2] Kasper B , Sleijfer S , Litière S , et al. Long‐term responders and survivors on pazopanib for advanced soft tissue sarcomas: subanalysis of two European Organisation for Research and Treatment of Cancer (EORTC) clinical trials 62043 and 62072. Ann Oncol. 2014;25(3):719‐724. 24504442 10.1093/annonc/mdt586 PMC4433518

[3] Ray‐Coquard I , Cropet C , Van Glabbeke M , et al. Lymphopenia as a prognostic factor for overall survival in advanced carcinomas, sarcomas, and lymphomas. Cancer Res. 2009;69(13):5383‐5391. 19549917 10.1158/0008-5472.CAN-08-3845 PMC2775079

[4] Sleijfer S , Ouali M , van Glabbeke M , et al. Prognostic and predictive factors for outcome to first‐line ifosfamide‐containing chemotherapy for adult patients with advanced soft tissue sarcomas: an exploratory, retrospective analysis on large series from the European Organization for Research and Treatment of Cancer‐Soft Tissue and Bone Sarcoma Group (EORTC‐STBSG). Eur J Cancer. 2010;46(1):72‐83. 19853437 10.1016/j.ejca.2009.09.022

[5] Stefanovski PD , Bidoli E , De Paoli A , et al. Prognostic factors in soft tissue sarcomas: a study of 395 patients. Eur J Surg Oncol. 2002;28(2):153‐164. 11884051 10.1053/ejso.2001.1242

[6] van der Graaf W , Orbach D , Judson IR , Ferrari A . Soft tissue sarcomas in adolescents and young adults: a comparison with their paediatric and adult counterparts. Lancet Oncol. 2017;18(3):e166–e175. 28271871 10.1016/S1470-2045(17)30099-2

[7] Vos M , Sleijfer S . EJC’s biennial report on metastatic soft tissue sarcoma: State of the art and future perspectives. Eur J Cancer. 2018;88:87‐91. 29202389 10.1016/j.ejca.2017.10.020

[8] Casali PG , Abecassis N , Bauer S , et al. Soft tissue and visceral sarcomas: ESMO‐EURACAN Clinical Practice Guidelines for diagnosis, treatment and follow‐up. Ann Oncol. 2018;29(Supplement_4):iv268‐iv269. 30285214 10.1093/annonc/mdy321

[9] Marulli G , Mammana M , Comacchio G , Rea F . Survival and prognostic factors following pulmonary metastasectomy for sarcoma. J Thorac Dis. 2017;9(Suppl 12):S1305‐S1315. 29119019 10.21037/jtd.2017.03.177 PMC5653498

[10] Eisenhauer EA , Therasse P , Bogaerts J , et al. New response evaluation criteria in solid tumours: revised RECIST guideline (version 1.1). Eur J Cancer. 2009;45(2):228‐247. 19097774 10.1016/j.ejca.2008.10.026

[11] Savina M , Le Cesne A , Blay J‐Y , et al. Patterns of care and outcomes of patients with METAstatic soft tissue SARComa in a real‐life setting: the METASARC observational study. BMC Med. 2017;15(1):78. 28391775 10.1186/s12916-017-0831-7 PMC5385590

[12] Blay J‐Y , van Glabbeke M , Verweij J , et al. Advanced soft‐tissue sarcoma: a disease that is potentially curable for a subset of patients treated with chemotherapy. Eur J Cancer. 2003;39(1):64‐69. 12504660 10.1016/s0959-8049(02)00480-x

[13] Lindner LH , Litière S , Sleijfer S , et al. Prognostic factors for soft tissue sarcoma patients with lung metastases only who are receiving first‐line chemotherapy: An exploratory, retrospective analysis of the European Organization for Research and Treatment of Cancer‐Soft Tissue and Bone Sarcoma Group (EORTC‐STBSG). Int J Cancer. 2018;142(12):2610‐2620. 29383713 10.1002/ijc.31286 PMC5947111

[14] Van Glabbeke M , van Oosterom AT , Oosterhuis JW , et al. Prognostic factors for the outcome of chemotherapy in advanced soft tissue sarcoma: an analysis of 2,185 patients treated with anthracycline‐containing first‐line regimens–a European Organization for Research and Treatment of Cancer Soft Tissue and Bone Sarcoma Group Study. J Clin Oncol. 1999;17(1):150‐157. 10458228 10.1200/JCO.1999.17.1.150

[15] Bui‐Nguyen B , Ray‐Coquard I , Chevreau C , et al. High‐dose chemotherapy consolidation for chemosensitive advanced soft tissue sarcoma patients: an open‐label, randomized controlled trial. Ann Oncol. 2012;23(3):777‐784. 21652583 10.1093/annonc/mdr282

[16] Verma S , Younus J , Stys‐Norman D , Haynes AE , Blackstein M , Members of the Sarcoma Disease Site Group of Cancer Care Ontario’s Program in Evidence‐Based Care . Meta‐analysis of ifosfamide‐based combination chemotherapy in advanced soft tissue sarcoma. Cancer Treat Rev. 2008;34(4):339‐347. 18313854 10.1016/j.ctrv.2008.01.005

[17] Blay JY , Bouhour D , Ray‐Coquard I , Dumontet C , Philip T , Biron P . High‐dose chemotherapy with autologous hematopoietic stem‐cell transplantation for advanced soft tissue sarcoma in adults. J Clin Oncol. 2000;18(21):3643‐3650. 11054437 10.1200/JCO.2000.18.21.3643

[18] Le Cesne A , Judson I , Crowther D , et al. Randomized phase III study comparing conventional‐dose doxorubicin plus ifosfamide versus high‐dose doxorubicin plus ifosfamide plus recombinant human granulocyte‐macrophage colony‐stimulating factor in advanced soft tissue sarcomas: A trial of the European Organization for Research and Treatment of Cancer/Soft Tissue and Bone Sarcoma Group. J Clin Oncol. 2000;18(14):2676‐2684. 10894866 10.1200/JCO.2000.18.14.2676

